# Impact of University Teachers’ Technological Training on Educational Inclusion and Quality of Life of Students with Disabilities: A Systematic Review

**DOI:** 10.3390/ijerph20032576

**Published:** 2023-01-31

**Authors:** José Fernández-Cerero, Marta Montenegro-Rueda, José María Fernández-Batanero

**Affiliations:** Department of Teaching and Educational Organization, University of Sevilla, 41004 Seville, Spain

**Keywords:** teacher training, digital competence, higher education, disability

## Abstract

Higher education institutions moving towards the inclusive education model have to offer quality education for all students. In this sense, the use of technologies favours not only the education of people with disabilities, but also their quality of life. However, these people may encounter real problems of access to technologies due, among other factors, to the lack of teacher training, causing a public health problem. In this line, our study includes a systematic review of the literature that aims to know the impact of the technological training of university teachers in relation to educational inclusion and the improvement of the quality of life of students with disabilities. To this end, a search of the literature published in the last decade was carried out in six databases (Dialnet, Google Scholar, Scopus, Web of Science, ERIC, and SciELO), selecting 14 articles out of 1204 initial ones. The studies were analysed following the PRISMA methodology. The main findings are the scarcity of research literature on the digital competence of university teachers and its impact on the educational inclusion and quality of life of students with disabilities. Likewise, there is a clear lack of knowledge about the use of digital tools and a lack of knowledge about the different disabilities that teachers may encounter in higher education classrooms.

## 1. Introduction

Higher education institutions moving towards the model of inclusive education are subject to a double and contradictory requirement: on the one hand, they have to offer quality education for all students, including those with disabilities, with the aim of avoiding exclusion, segregation, and discrimination, facilitating an improvement in their quality of life; and on the other hand, they have to recognise and respond in an adjusted manner to the individual characteristics of each student in order to favour and facilitate their educational inclusion.

There is a meeting between the contributions of the concept of quality of life and the approaches of inclusive education as the most appropriate model to achieve quality education for all students. On the other hand, the use of technologies favours not only the education of people with disabilities, but also their quality of life [1]. Technologies allow us to communicate and get by in our daily lives. In this sense, teachers have an important role to play in favouring the social and digital inclusion of people and, above all, of students with disabilities, who encounter greater difficulties in the education system. To this end, we believe that greater digital competence is necessary for teachers to make good use and mastery of these technological tools, contributing to improving the quality of life of students [2].

Considering the role played by information and communication technologies (ICTs) in today’s society, it is essential to highlight this concept, as it has been referred to from different perspectives in specialised research. Thus, from a technical point of view, ICT is considered as any digital tool that we use to work with information [3]. From an institutional perspective, it is defined as those electronic resources that transmit information [4]. Finally, from an educational point of view, ICTs are any medium, resource, technical tool, or device that favours and develops information, communication, and knowledge [5].

With regard to competences, these form the set of resources (knowledge, skills, and attitudes) that teachers need to satisfactorily resolve the situations they face in their professional work. In this sense, digital competence is a key concept for digitised higher education institutions. Several notions are used to refer to the same concept, such as digital competences [6], digital skills [7], and digital literacy [8]. Teachers’ digital competence is often described and defined in terms of practical use. Along these lines, the European Parliament [9] defined digital competence as involving the critical and confident use of information society technologies for work, leisure, and communication. More recently, authors such as Suárez-Rodríguez, Almerich, Díaz-García and Fernández-Piqueras [10] understand ICT competences on the part of teachers as “the set of knowledge and skills necessary for them to use these technological tools as more integrated educational resources in their daily practice”.

With regard to the term disability, we must clarify that in our study, we understand it as any form of impairment or limitation in a person’s normal functioning [11]. These limitations can be generated by environmental barriers that prevent the participation and inclusion of these individuals, where technological advances allow these inaccessible environments to be modified.

Quality of life is a concept that differs, including or excluding factors, depending on the organisation that defines it. It is a complex term that reflects social and individual aspects (objective and subjective) and multiple spheres such as physical, emotional, educational, and occupational. In our case, we refer to that established by the World Health Organisation [12], which defines it as the individual’s perception of his or her position in life in the context of the culture and value system in which he or she lives and in relation to his or her goals, expectations, standards, and concerns.

Following this line, we believe that this study is very useful to assess the current situation in terms of research on teacher training in ICT applied to students with disabilities. For all these reasons, it has been decided to carry out an exhaustive review of the current publications on university teacher training in ICT, because the correct training of university teachers in digital competences is reflected in an improvement in the teaching and learning processes of students [13].

## 2. Research in University Teacher Training in ICT and Disability

In the Spanish university environment, studies on ICT in general (technological competencies of teachers, technological competences of students, technologies to support learning, etc.) are relatively abundant [14], but studies in relation to technology and disability in higher education are very limited. Emphasising inclusion and equity as the foundation for quality education and learning requires not only the need for the removal of architectural barriers, but also virtual spaces and processes. Thus, university systems need to review their practices to ensure the learning and participation of all students. In the special case of students with disabilities, many authors have identified the obstacles they face at university [15,16,17], where classroom practices are identified as the main difficulty of permanence.

In relation to teachers, there is a growing concern about their technological training to enable them to improve the experiences of these students through ICT [18]. Technological training is one of the main barriers that teachers encounter when responding to the needs of students with disabilities [19,20,21]. Moreover, authors such as Moriña, Molina, Melero, and Carballo [22] conducted a study with the aim of finding out more about the needs that higher education teachers have to improve the inclusiveness of students with disabilities. It was concluded that teaching staff did not have the necessary knowledge to promote student inclusion, nor were they aware of the needs of students with disabilities.

These needs for technological teacher training in aspects related to the attention to student diversity have also been highlighted in the health crisis that has led to COVID-19, where factors such as the age of the teaching staff are a handicap when it comes to facing the technological challenges that ICT has presented us with. This is highlighted in several recent studies and is associated with mental health problems [23].

In short, higher education institutions are aware of the positive impact that an effective integration of these tools can have as support for students with disabilities [24], but there are other limitations, such as the scarcity of resources [25] or the lack of government funding and the economic problems of university institutions [26,27].

It is in this context that we set out the following study with the intention of further investigating teacher training and technology education with respect to student inclusion and consequently improving their quality of life.

## 3. Purpose and Research Question

The main objective of the systematic literature review study is to learn about the technological training of university teachers in relation to educational inclusion and the improvement of the quality of life of students with disabilities, as well as to identify future directions of research on such training.

In this line, we intend to answer the following research questions (RQ):

RQ1: What is the current state of research on university teacher training in information and communication technologies (ICTs) as a support for students with disabilities?

RQ2: Do university teachers have the necessary technological competences for educational inclusion and improvement of the quality of life of students with disabilities?

RQ3: What are the barriers to teacher training for improving inclusion in university classrooms?

RQ4: What are the future directions of research focused on the role of teachers in the inclusion of technologies to support students with disabilities?

## 4. Methodology

To address the research questions posed, we conducted a systematic review to examine published empirical research on university teacher training in ICT as a support for students with disabilities. We used the PRISMA statement to guide the article selection process for the systematic review [28].

### 4.1. Search Criteria

Prior to the search for the articles that will define our results according to the subject matter, a series of inclusion and exclusion criteria were formulated that would allow us to delimit the results obtained and that could be useful for the research.

#### 4.1.1. Inclusion Criteria

- Empirical research conducted during the last decade (2012–2022).
- Research conducted in English or Spanish.
- Research published in book chapters or peer-reviewed journals.
- Research dealing with the training of university teaching staff in the use of ICT to support students with disabilities.

#### 4.1.2. Exclusion Criteria

Following the establishment of the inclusion criteria, it is necessary to note certain aspects that we did not take into account when including studies in the literature review:

- Literature review research.
- Research in the form of conference proceedings, doctoral theses, or technical reports.
- Research that is not in the established time interval (2012–2022).
- Research that is in a language other than English or Spanish.
- Research that is not related to the subject to be studied.
- Research that is duplicated.

### 4.2. Literature Search

We conducted a search using the following databases: Dialnet, Google Scholar, Scopus, Web of Science (WoS), Education Resources Information Center (ERIC), and SciELO. These 6 databases were selected because of their great importance in the field of social sciences, more specifically in the field of education. The literature search focused on research published between 2012 and 2022. This time interval was selected because scientific production in this field of study has increased over the last decade due to the importance of technologies in education. Only studies published in English or Spanish were selected, because English is the language with the highest scientific production and Spanish is the language with the highest scientific production, and Spanish is the language with the highest scientific production for a better approach to our context in the field of study.

For the search, synonyms for the terms ‘teacher education’, ‘ICT’, ‘digital competence’, ‘higher education’, and ‘disability’ were used. This resulted in the following Boolean search equation: (“teacher education”) AND (“ICT” OR “technology”) AND (“digital competence” OR “digital skill” OR “technology skill”) AND (“higher education” OR “university”) AND (“disability” OR “inclusion” OR “diversity” OR “quality of life”). A total of 1204 results were identified in the initial search (last search date: 9 November 2022). To reduce the number of articles, we adapted our string to consider only studies that include these terms in the title, abstract, and/or keyword fields, yielding a total of 534 studies in the total of the six databases analysed. After eliminating 173 duplicate studies, we were left with 361 for further review before selecting studies for review.

### 4.3. Study Selection

**Figure 1:**
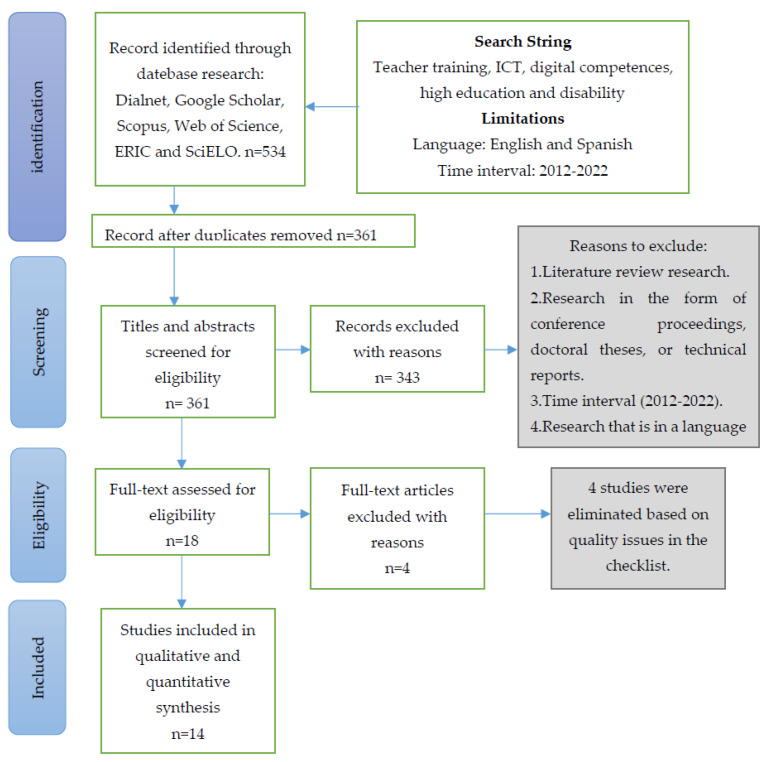
Flow chart of the search, selection, and inclusion of studies.

All identified articles (*n* = 361) were screened by the researchers for initial selection according to inclusion and exclusion criteria. This resulted in an exclusion of 343 publications that did not meet these criteria. The 18 selected articles were read in full text and assessed for eligibility based on their methodological quality using a checklist. Only articles that met at least seven of the assessment criteria were accepted in our review. In total, 14 were included in our quantitative and qualitative synthesis. Figure 1 summarises the steps in the process of study identification, selection, and inclusion.

### 4.4. Assessment of the Methodological Quality of the Studies

The methodological quality of the 18 identified articles was assessed using Johanna Briggs’ checklist (JBI), where it was examined by independent critical review (eleven-point checklist) [29]. For the internal assessment of the quality of the proposed study, the checklist was assessed by two independent researchers (Appendix A) to the research in a masked form in order to avoid assessment bias by the authors themselves. The checklist included the following assessment criteria:

Is the purpose of the research clearly specified?

Does it specify the type of technology applied in the study?

Did the sample use only university teachers?

Does it specify the level of teacher education?

Does it analyse different types of disabilities?

Are the data extraction instruments appropriate?

Are the results obtained useful for the scientific community?

Are the authors’ conclusions based on the data analysed?

Are recommendations made for future research?

Four studies [30,31,32,33] were excluded based on the quality issues raised in the checklist.

### 4.5. Data Analysis and Extraction

With the aim of answering the research questions posed above, we set out to carry out a content analysis divided into a qualitative and a quantitative methodology of the 14 research studies we obtained after sifting through the selected criteria. In the quantitative analysis, we developed a series of graphs that allowed us to gain a deeper understanding of the general aspects of the subject in question. For the qualitative analysis, the VOSviewer tool was used to categorise the main themes of the study in question.

For data extraction, a table was created with all the information on the articles included in the review (authors, year of publication, methodology used, type of technology, disability, level of teacher training, and main obstacles) ordered alphabetically according to the surname of the first author (Table 1).

## 5. Results

In the following, this study will share the results in two separate sections. The first relates to the quantitative results concerning the data relating to the general description of the studies analysed. In a second part, the qualitative results collected through a network map of keyword co-occurrence are presented.

**Figure 2:**
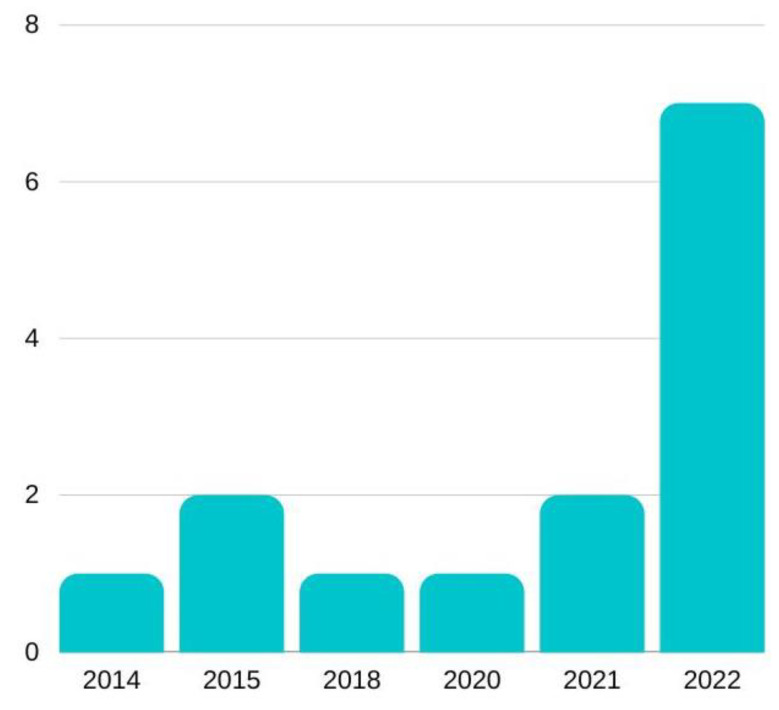
Distribution of studies by year of publication.

In the study carried out on the “Impact of technological training of university teachers on the educational inclusion and quality of life of students with disabilities”, a total of 14 articles found in different databases published over the last decade, i.e., from 2012 to 2022, were selected. All the articles collected are closely related to the proposed topic and are considered key studies for obtaining greater knowledge of the field of research. Figure 2 shows the distribution of the studies selected in the literature review by year of publication. It can be seen that the largest number of articles published on the subject is in 2022, revealing that the importance of the subject is growing despite the scarcity of research on the subject.

**Figure 3:**
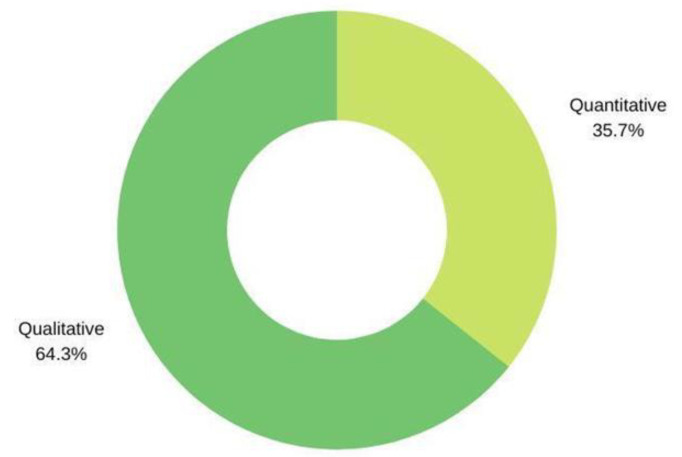
Distribution of studies by methodology used.

Figure 3 shows the distribution of the studies compiled in the literature review according to the methodology used by the authors. At first glance, it can be seen that the qualitative methodology accounts for 64.3% of the studies. In contrast, the quantitative methodology accounts for 35.7% of the selected studies. On the other hand, none of the 14 studies collected used a mixed methodology. Following this line, we can consider that qualitative methodology is mostly applied in this area.

**Figure 4:**
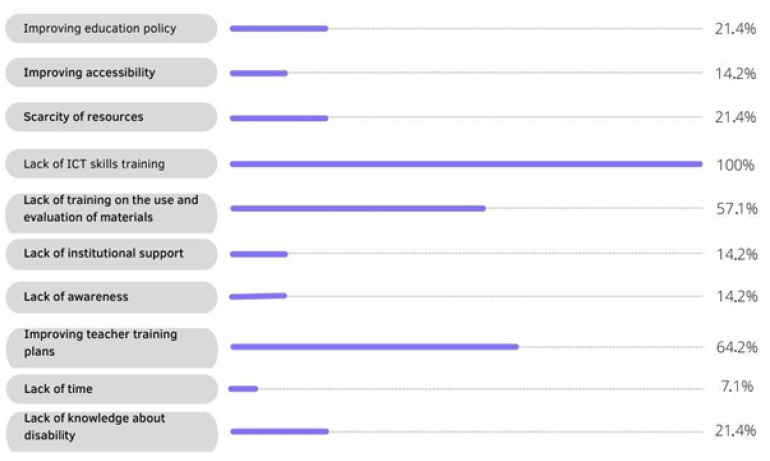
Quantitative analysis of obstacles.

Figure 4 shows a quantitative analysis of the barriers observed in the reading of documents through the previously selected databases. In the first instance, it should be noted that a total of 10 barriers were found, these being the main obstacles to be highlighted. It can be seen that 100% of all the studies mention the low qualification of higher education teachers in digital competences to support students with disabilities, and that urgent training is needed to improve the teaching and learning process for all students. The need to improve teacher training plans is another of the obstacles to be highlighted (64.2%), as this is closely linked to teacher training, with the improvement of one being inevitable in order to establish progress in the quality of education. On the other hand, 57.1% of the studies mention the lack of teacher training on the use and evaluation of materials, as teachers do not know how to apply digital tools and how to evaluate pupils with disabilities. In the sample, 21.4% consider that the lack of resources on the part of the institution, the need to reform educational policies to achieve inclusivity, and teachers’ lack of knowledge about disability are among the main barriers that prevent the inclusion of students in education. Thus, 14.2% of the authors refer to the importance of improving accessibility, the lack of awareness, and the lack of support from institutions. Last but not least, lack of teacher time is mentioned as one of the keys to not acquiring the digital teaching skills needed to support students. All these barriers represent a major handicap that prevents students with some kind of limitation from being properly integrated into university classrooms, and it is considered inevitable that they need to be broken down in order to achieve equal quality education for all students.

**Figure 5:**
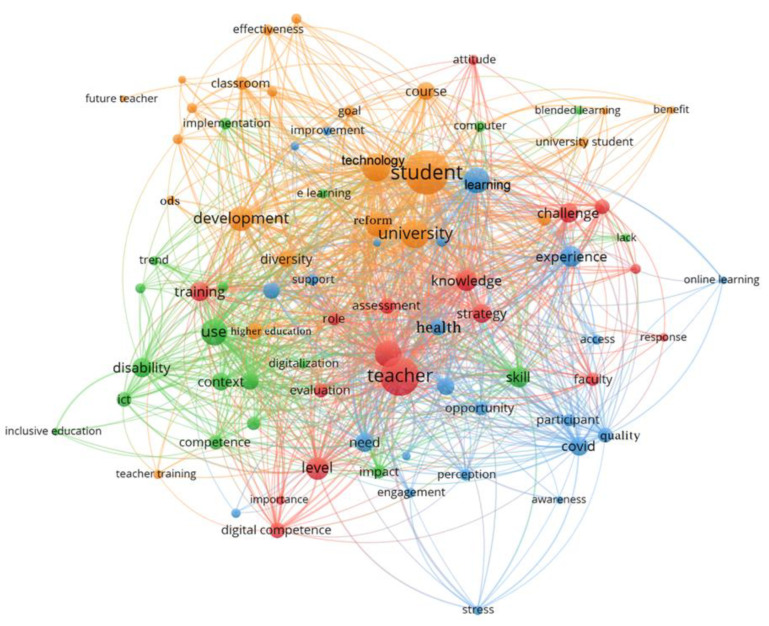
Keyword co-occurrence map.

Then, from the analysed references, the co-occurrence of keywords was analysed using the VOSviewer software [46]. Keyword co-occurrence analysis makes it possible to identify how two or more terms are related to each other and to reveal the critical points of a particular field of research. Figure 5 presents the joint word network or keyword co-occurrence:

Each node in the network represents a keyword, the size indicating the number of times it is repeated [47]. Each colour represents a thematic group (cluster). In this study, a total of 65 keywords were retrieved and grouped into 4 clusters (red, green, blue, and orange) to identify the key themes of the research. Table 2 lists the keywords included in each cluster, as well as the themes related to each of the studies analysed.

## 6. Discussion

The results presented in the above selection are discussed with respect to the research question in the study:

*Q1: What is the current state of research on university teacher training in information and communication technologies as a key support for students with disabilities?*

After carrying out the bibliographical review of the literature, we can highlight, firstly, the scarcity of studies on the subject, as only a total of 14 studies on higher education have been carried out in the last 10 years, studies that coincide with various authors [48]. This fact is quite remarkable, as throughout the review, the importance of ICT competences in the field of education has been highlighted, not only for the improvement of inclusion and quality of life of students, but also for the improvement of teaching and learning processes at all educational levels [49]. Secondly, the superiority of the use of qualitative methodology over quantitative methodology has been highlighted, suggesting that this type of methodology could be more beneficial if we aim to obtain more detailed information about a subject in a given context [50].

*Q2: Do university teaching staff have the necessary technological skills for educational inclusion and improving the quality of life of students with disabilities?*

In response to the second research question, the acquisition of digital competence in university teaching staff is of vital importance, as it is a magnificent tool to allow greater accessibility to both institutions and content for all students. The Common Framework for Digital Competence in Education [51] is an adaptation of the European Framework of Digital Competence for Citizens and the European Framework of Digital Competence for Educators, which is divided into six modules: professional engagement, digital content, teaching and learning, assessment and feedback, student empowerment, and development of students’ digital competence. Following this line, several authors who have carried out studies on this subject reflect that teachers do not have these necessary competences, as it turns out that they are not technologically prepared to support students with disabilities [27,35].

Therefore, digital competence is postulated as one of the most necessary skills for teachers in the 21st century, as it is necessary to be able to continue developing and using these skills in university classrooms, allowing them to contribute to a better educational quality, both in students and in the education system, and to be able to continue adapting to the new needs that arise in society, as well as the educational challenges to arise [52].

*Q3: What are the barriers to teacher training for improving inclusion in university classrooms?*

In response to the third research question, the use of ICT can be tools that can benefit all students, regardless of their educational level, as studies confirm that they are capable of eliminating obstacles that, at first glance, are totally detrimental to improving educational quality [53]. Thus, the vast majority of the research compiled in the literature review shows the main barriers that students with disabilities may encounter when accessing higher education, which are an impediment to achieving student inclusion [54].

Numerous authors highlight the scarce training of university teaching staff in technological competences, considering the teacher as the key element when it comes to removing obstacles and impediments [22,55,56]. Moreover, the educational plans and policies that aim to remedy this situation in university institutions in Spain tend to be more integrative than inclusive [57]. On the other hand, although institutions have the necessary technological tools to support students with disabilities and are accessible, in most cases they are not being used appropriately, preventing them from being effective and useful [58].

*Q4: What are the future directions of research focusing on the role of teachers in the inclusion of technologies with students with disabilities?*

Based on the analysis carried out using the VOSviewer software, a bibliometric analysis was presented visualising the research trends concerning digital teacher education in higher education. This analysis showed as a result the emerging lines of research and research directions through four clusters that group the keywords contained in the titles and abstracts of the studies in the databases. We can therefore establish that there are four main lines of study.

The first is focused entirely on the need to improve the digital training of teachers. The figure of the teacher is fundamental to apply ICT in their teaching practice, as well as to promote new forms of teaching [34]. The second is a focus on the transformations and advances in society that are giving rise to the need for the implementation of ICT with students with disabilities in higher education [39]. The third is with the importance of promoting the use of technologies as fundamental support tools to improve the quality of life of students with disabilities [38,59]. Finally, the last one is the development of educational policies that favour the implementation of ICT in higher education. Access opportunities for students with disabilities to use ICTs is one of the factors that causes the digital divide, given that it limits equal opportunities in access to information and knowledge [37].

Thus, attention should be paid to the aforementioned lines of research, as they are the focus of most of the research that is seeking to improve the training of university teachers in the use of ICT to support students with disabilities.

## 7. Conclusions

The systematic review of the literature has shown that teacher training in digital competences to support students with disabilities to improve their quality of life is scarce, as there is a clear lack of knowledge about the use of digital tools and a lack of knowledge about the different disabilities that teachers may encounter in higher education classrooms. This demand should be made relevant in today’s society by offering a wide range of diverse courses and training experiences to meet the specific needs of individuals.

Teachers have not obtained the necessary competences to achieve the inclusion of all students, being the main responsible in the educational process. This is accompanied by certain obstacles that support this situation, which must be resolved as soon as possible in order to improve education, such as the non-existence of specific educational plans and the scarcity of training plans in the field of research.

Information and communication technologies are inclusive and accessible to the individual as long as they are used successfully. On the other hand, a bad use of these tools can produce the opposite effect, a marginalisation of this group in the classroom. However, we cannot ignore the enormous possibilities and benefits they offer, not only to students with disabilities, but also to other individuals, resulting in a better quality of life for the person. It has been established that the need for specific training has become one of the great challenges in higher education institutions, as well as an improvement in the accessibility and availability of resources to individuals.

### Limitations

When carrying out the systematic review of the literature, a series of limitations were found in the process. The first of these is closely related to the scarcity of studies that have been found after searching the different databases, as there are not many authors who carry out a similar study in the field of higher education. On the other hand, it is worth highlighting the scarce evidence available on the corroboration of the factors that cause the lack of digital training of teaching staff.

## Figures and Tables

**Table 1:** Description of the studies included in the review.

Authors	Year	Method	Type of Technology	Type of Disability	Level of Training	Main Obstacles.
Aranda et al. [34]	2014	Qualitative	Screen readers, computers, e-books, tablets	Physical, visual, hearing disability	Low	2, 3, 5, 4
Cabero et al. [14]	2022	Quantitative	Not specified	Motor, cognitive, visual, hearing disability	Low	8, 1, 4
Fernández Batanero et al. [35]	2022	Quantitative	Not specified	Motor, cognitive, visual, hearing disability	Low	4, 10, 8, 5
Fernández Batanero et al. [36]	2022	Quantitative	Not specified	Motor, cognitive, visual, hearing disability	Low	5, 4,
Huamán et al. [37]	2022	Quantitative	Not specified	Motor, cognitive, visual, hearing disability	Low	4, 5, 1,
Kapalu et al. [38]	2021	Qualitative	Audio Recorded lessons, JAWS, Social media platforms, Computers installed with signing software and sign language video lessons and e-learning platforms	Motor, cognitive, visual, hearing disability	Low	2, 4, 5, 8
Leiva et al. [39]	2022	Qualitative	Not specified	Motor, cognitive, visual, hearing disability	Low	8, 4
López et al. [40]	2018	Qualitative	Speech synthesiser, screen reader, Braille keyboard	Visual disability	Low	8, 4, 6, 3
Medina et al. [41]	2021	Quantitative	Not specified	Motor, cognitive, visual, hearing disability	Low	4, 6, 7
Moriña et al. [42]	2015	Qualitative	Not specified	Motor, cognitive, visual, hearing disability	Low	4, 8
Moriña et al. [22]	2015	Qualitative	Not specified	Motor, cognitive, visual, hearing disability	Low	4, 1, 8, 5
Perera et al. [43]	2022	Qualitative	Not specified	Motor, cognitive, visual, hearing disability	Low	4, 5, 8, 10
Román et al. [44]	2022	Qualitative	Not specified	Motor, cognitive, visual, hearing disability	Low	4, 8, 9, 7
Román et al. [45]	2020	Qualitative	Mobile applications	Cognitive	Low	3, 4, 5, 10

Note: 1. Improve educational policy; 2. Improve accessibility; 3. Shortage of resources; 4. Lack of teacher training in ICT skills; 5; 5. Lack of teacher training in the use and evaluation of materials; 6. Lack of institutional support; 7. Lack of awareness raising; 8. Improve teacher training plans; 9. Lack of time; 10. Lack of knowledge about disability.

**Table 2:** Description of the keywords included (clusters) and related topics in each study.

Authors	Themes Included (Cluster)			
	Teacher Training (Red)	Technologies and Disabilities (Green)	Quality of the Students (Blue)	Education Policy (Orange)
	teacher, level, digital competence, importance, evaluation, role, assessment, strategy, faculty, response, challenge, training, attitude	ICT, computer, blended learning, lack, skill, impact, digitalization, competence, context, use, disability, inclusive education, trend, implementation, e-learning	health, opportunity, perception, engagement, need, stress, awareness, COVID, learning, participant, quality, access, experience, online learning, improvement, support	student, university, technology, development, diversity, reform, higher education, university student, learning, benefit, course, goal, SDG, teacher, training, future training, effectiveness, classroom
Aranda et al. [34]		x	x	
Cabero et al. [14]	x	x	x	x
Fernández Batanero et al. [35]	x	x		
Fernández Batanero et al. [36]		x		
Huamán et al. [37]		x		x
Kapalu et al. [38]	x	x	x	
Leiva et al. [39]	x	x		
López et al. [40]	x	x		x
Medina et al. [41]		x	x	x
Moriña et al. [42]	x	x		
Moriña et al. [22]	x	x		x
Perera et al. [43]	x	x		
Román et al. [44]	x	x	x	
Román et al. [45]		x		

The X means that it meets the marked characteristics.

## Data Availability

Not applicable.

## Appendix A

**Table A1:** Checklist.

	Q1	Q2	Q3	Q4	Q5	Q6	Q7	Q8	Q9
Aranda et al. [34]									
Cabero et al. [14]									
Batanero et al. [33]									
Batanero et al. [35]									
Batanero et al. [36]									
Huamán et al. [37]									
Kapalu et al. [38]									
Leiva et al. [39]									
López et al. [40]									
Medina et al. [41]									
Moriña et al. [22]									
Moriña et al. [42]									
Okolo et al. [31]									
Perera et al. [43]									
Rojas et al. [32]									
Román et al. [44]									
Román et al. [45]									
Rosario et al. [30]									

The green color meets the marked characteristics. The red color means that it does not meet the marked characteristics.
